# Educating communities to improve health in Ethiopia

**DOI:** 10.2471/BLT.16.020516

**Published:** 2016-05-02

## Abstract

The health of women and children has improved considerably in rural Ethiopia, thanks to the dedication of its health extension workers and its network of volunteers. Bethlehem Kiros reports.

“I wouldn’t be here if it weren’t for her,” says Tejitu Wondimu, a mother of six, gesturing towards Adanech Tsega, a community health worker in the Amhara region of Ethiopia.

In 2013, Tejitu was planning to give birth at a health centre, but she went into labour early and gave birth in the early hours of the morning at her home in the village of Yetsed.

A relative went to Adanech’s house to tell her. Adanech arrived soon afterwards only to find the baby dead and Tejitu unconscious and bleeding.

“The family insisted that she was sleeping, but I knew she’d passed out,” Adanech recalls. After an argument with Tejitu’s father-in-law, Adanech persuaded some people in the village to help her take Tejitu to the health centre.

“The district ambulance was out of service so we carried her on a traditional stretcher to the roadside,” says Adanech, referring to an improvised construction of planks and rope used in rural areas to carry people to hospital. That day Adanech’s persistence saved Tejitu’s life.

“The first thing I saw when I woke up was Adanech sitting next to my bed,” says Tejitu. “Later my family told me she had jumped in front of a vehicle and begged the driver to take me to the health centre.”

Adanech is one of about 38 000 community health workers at the heart of Ethiopia’s flagship Health Extension Programme and on the frontline of the country’s efforts to reduce maternal and child mortality and improve people’s health in general.

These health workers are from the communities that they serve. They are trained to educate people about health and deliver basic health care, including vaccinations, but do not have formal medical qualifications.

Many have stories of heroism to tell, as well as of everyday grind.

Adanech must walk to homes all over the rural sub-district from her health post in Yetsed village and, when she arrives, her new ideas are not always welcome.

“Sometimes after walking for hours to get to a village, we find the same problems that we thought we’d already addressed during previous visits.

“But there is less resistance today as people are more aware of the need for health care after years of door-to-door education,” Adanech says.

“People are more aware of the need for health care after years of door-to-door education.”

Adanech Tsega

Ethiopia has created an innovative volunteer system known as the Women’s Development Army with its own structure and terminology. “In each village, groups of five women volunteers make up a larger group of 30 led by a woman from a ‘model’ household. We train these volunteers and they assist us by keeping an eye out for new pregnancies in their neighbourhood,” says Adanech.

“Once a pregnant woman has been identified, we visit her and hold a ‘family meeting’ with the husband and other decision-makers, like parents and in-laws, to discuss the need for antenatal care and skilled birth attendance,” she says. “This avoids disagreements later on.”

In 1990, an estimated 1250 women died for every 100 000 live births in Ethiopia. It was one of the highest maternal mortality rates in the world at the time. Since 1990, maternal mortality has fallen by 71% to 353 per 100 000 live births, just four percentage points short of the millennium development goal (MDG) target.

“This success can mainly be attributed to the fact that we have a strong health extension programme that is firmly rooted in the community,” says Dr Ephrem Tekle, Director for the Maternal and Child Health Directorate in the health ministry in the capital Addis Ababa.

“But there are other factors. Many mothers used to die because they gave birth at home. Today more mothers are coming to health facilities to give birth,” he says, stressing the important role community health workers play in health education and support for these mothers.

Ephrem adds that 68% of births were assisted by skilled birth attendants between July 2015 and February this year. In rural areas these are midwives, in urban areas, they may be midwives, nurses or physicians. “The increase in skilled birth attendance in our health facilities has also contributed to the reduction in maternal deaths,” Ephrem says.

Ethiopia launched its health extension programme in 2003 and started building a new cadre of salaried community health workers, like Adanech, to attend to the health needs of its rural population who, today, account for 80% of the country’s 91 million people. The vast majority, about 33 000, of health extension workers are based in rural communities.

Over the last two decades, child survival has also improved. Child mortality fell by two thirds between 1990 and 2012, achieving MDG 4 three years ahead of the target.

“By 2014, the under-five mortality rate had reached 59 per 1000 live births, which represents a decline of 71% compared to 205 per 1000 live births in 1990,” says Dr Hailemariam Legesse, who coordinates child health programmes at the United Nations Children’s Fund in Ethiopia.

For Hailemariam, child survival has improved largely because health extension workers are trained to identify and treat major childhood killers such as pneumonia, diarrhoea, malnutrition and newborn sepsis.

“Still, nearly 180 000 children die from preventable or treatable conditions every year,” he says, adding that he expects further improvements in child health, now that health extension workers are trained in the integrated community case management of childhood illnesses, referring to Ethiopia’s adaptation of WHO’s Integrated Management of Childhood Illness guidelines.

Dembecha, where Adanech works, is one of Ethiopia’s high performing districts when it comes to maternal and child health. The hope is that other districts will follow its example.

“We visit every household in our *kebele* to do health promotion,” says Adanech, referring to Ethiopia’s smallest administrative unit. “We do our best to persuade mothers to deliver their babies in health centres and to get sick children the appropriate medical attention.”

Dembecha Health Bureau, the district’s administrative centre for health, is run by Anteneh Jember who is responsible for six health centres, including Yetsed village where Adanech works.

“Although we worked intensively last year, out of the 5089 women expected to give birth, only 3605 of them delivered at health centres (71%) and two mothers died during home delivery,” Anteneh says.

“At the beginning of this year, we identified the problems, decided how to resolve them and we are now implementing the solutions.” The solutions included stepping up efforts to identify more expectant mothers, and hiring more midwives and training them in compassionate and respectful care. As a result, since July last year more births (3277 out of 3455) have taken place at a health facility than the previous year, and only 5% of births were at home, he says.

In Yetsed village, where Adanech is one of two health extension workers covering the sub-district of 5300 inhabitants, a midwife is always present at monthly meetings with pregnant women at the local health post to answer questions, provide education and give them their due dates. “We serve them coffee to put them at ease, so that they can ask questions about their pregnancies and talk about the challenges they are facing,” says Adanech.

When women from remote places complete their eighth month, they are invited to come to the district health centre to wait for the birth. If they don’t show up, a health worker goes to their home to encourage and help them to come.

For Ephrem, one of the greatest challenges is to scale up these strategies across the whole country. “If you take the Somali region, for example, only 10% of births are assisted by skilled attendants,” he says, noting the gap in health service delivery to nomadic communities.

The health ministry is now developing a mobile health extension programme, so that the health worker travels with the community. The idea is to store the vaccines in refrigerators that are solar powered and that can be carried on camels’ backs, Ephrem says.

Ethiopia’s efforts to improve maternal and child health are part of the country’s Health Sector Transformation plan, launched in October 2015, that has set goals to increase health service utilization and equity in service provision, and improve the quality of health care at all levels.

According to Ephrem, as in the case of the MDGs, there is a strong political will to prioritize maternal, newborn and child health and achieve the sustainable development goal to end all preventable maternal and child deaths by 2030, one of the new United Nations development goals set in September 2015.

 “It requires quite a commitment at all levels. We are determined to halve maternal mortality in the next five years to achieve our 2030 vision that reflects the sustainable development goal targets,” Ephrem says.

**Figure:**
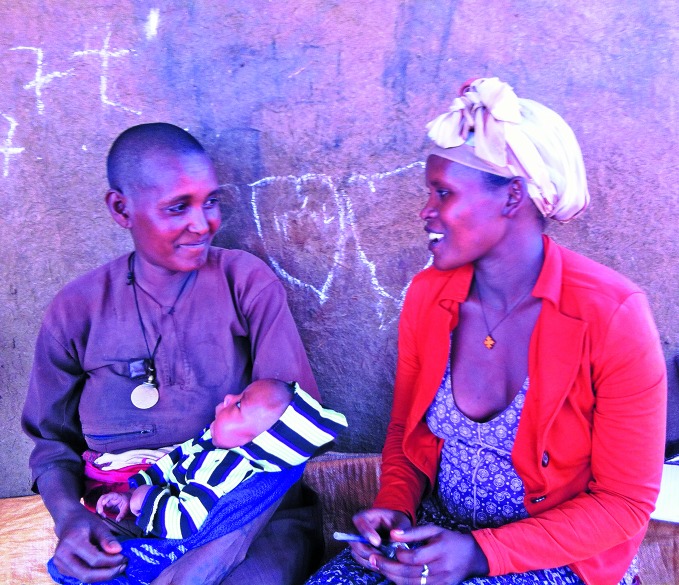
Tejitu Wondimu, mother of six children, and Adanech Tsega, health extension worker, sit and talk outside Tejitu’s house

WHO/ Bethlehem Kiros

**Figure:**
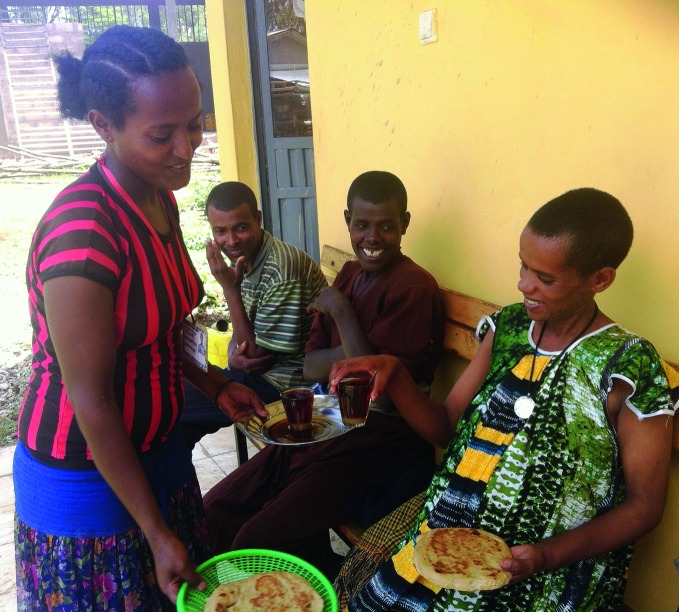
Expectant mothers are served food in the maternity waiting ward in Dembecha Health Centre

WHO/ Bethlehem Kiros

